# Supplementary material for: Splice-Junction-Based Mapping of Alternative Isoforms in the Human Proteome
Source: Cell Rep. Author manuscript; Available in PMC 2020 Jan 15. (PMC6961840; doi:10.1016/j.celrep.2019.11.026)
Supplement: 3 [file NIHMS1546469-supplement-3.zip › DF2/PXD009737/TestisGluC-84-Q9UM54-AQADLALRRGPAVLATKAAAGTKKYD.pdf]

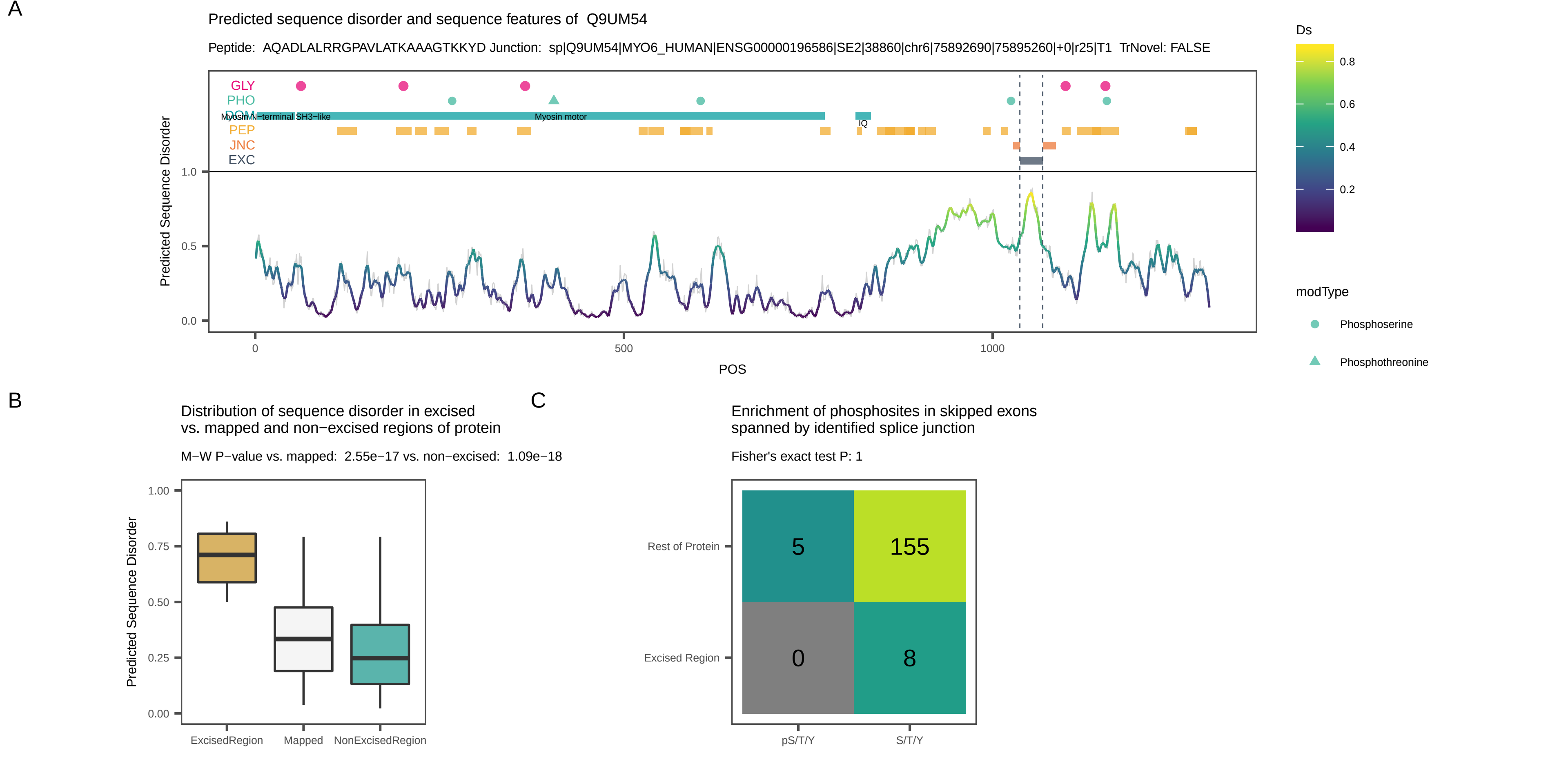
A
Predicted sequence disorder and sequence features of Q9UM54
Peptide: AQADLALRRGPAVLATKAAAGTKKYD Junction: sp|Q9UM54|MYO6_HUMAN|ENSG00000196586|SE2|38860|chr6|75892690|75895260|+0|r25|T1 TrNovel: FALSE
GLY
PHO
DOM
Myosin N−terminal SH3−like
Myosin motor
IQ
PEP
JNC
EXC
Predicted Sequence Disorder
1.0
0.5
0.0
0
500
1000
POS
Ds
0.8
0.6
0.4
0.2
modType
Phosphoserine
Phosphothreonine
B
Distribution of sequence disorder in excised vs. mapped and non−excised regions of protein
M−W P−value vs. mapped: 2.55e−17 vs. non−excised: 1.09e−18
Predicted Sequence Disorder
1.00
0.75
0.50
0.25
0.00
ExcisedRegion
Mapped
NonExcisedRegion
C
Enrichment of phosphosites in skipped exons spanned by identified splice junction
Fisher's exact test P: 1
Rest of Protein
Excised Region
5
155
0
8
pS/T/Y
S/T/Y
